# *Lycium ruthenicum* Polysaccharides Alleviate CCl_4_-Induced Acute Liver Injury Through Antioxidant and Anti-Inflammatory Effects

**DOI:** 10.3390/nu17213359

**Published:** 2025-10-25

**Authors:** Jie Xiao, Chunpeng Li, Yuxuan Pei, Shuhua Xu, Haotian Zhao, Wen Xiang, Jiayi Wei

**Affiliations:** 1 Department of Recreation and Sports and Health Tourism, Russian Sports University, Moscow 105122, Russia; a13837338157@163.com (J.X.); lichunpeng61@gmail.com (C.L.); 2 School of Food Science and Technology, Jiangnan University, Wuxi 214041, China; peiyuxuan0127@126.com (Y.P.); 6230112100@stu.jiangnan.edu.cn (S.X.); 1120200668@mail.nankai.edu.cn (W.X.)

**Keywords:** *Lycium ruthenicum* polysaccharide, acute liver injury, anti-inflammatory, antioxidant

## Abstract

[Introduction] The study aimed to investigate the protective effect of *Lycium ruthenicum* polysaccharides (LRPs) against carbon tetrachloride (CCl_4_)-induced acute liver injury (ALI). [Method] After LRP was extracted and characterized, a CCl_4_-induced cell damage and mouse ALI model was established to evaluate its anti-inflammatory and antioxidant capacities. [Results] The results demonstrated that LRP markedly attenuated hepatocyte necrosis, alleviated cellular edema and degeneration, and preserved nuclear integrity to sustain hepatic function, thereby restoring hepatic architecture. It downregulated serum ALT and AST levels, reduced MDA content in liver tissue, and enhanced SOD activity. Additionally, LRP inhibited the expression of pro-inflammatory cytokines TNF-α and IL-6, while upregulating the anti-inflammatory cytokine IL-10. [Conclusions] These findings suggest that LRP effectively alleviates CCl_4_-induced ALI through both antioxidant and anti-inflammatory effects, demonstrating its potential as a novel liver-protective agent.

## 1. Introduction

Acute liver injury (ALI) is a severe clinical condition triggered by drugs, viruses, or toxins, characterized by rapid hepatocyte necrosis leading to liver dysfunction or failure [1,2,3]. Epidemiological data worldwide indicate that liver disease-related mortality remains high in the global disease burden [4]. Current clinical interventions face significant challenges. Synthetic drugs are often associated with side effects including hepatotoxicity [5,6], nephrotoxicity, and bone marrow suppression, with prolonged treatment durations and high costs. These limitations underscore the urgent need to develop novel therapies that are both effective and exhibit low toxicity.

The progression of liver injury is primarily driven by a synergistic amplification of inflammatory responses and oxidative stress. Hepatocyte damage leads to excessive activation of Kupffer cells, which release pro-inflammatory mediators such as tumor necrosis factor-α (TNF-α) and interleukin-6 (IL-6), thereby triggering neutrophil infiltration and initiating a pro-inflammatory cascade [7,8]. Concurrently, oxidative stress is characterized by the rapid accumulation of reactive oxygen species (ROS), resulting in the depletion of superoxide dismutase (SOD) and elevated levels of the lipid peroxidation product malondialdehyde (MDA) [9,10]. Notably, these two pathways interact through key molecular nodes forming a positive feedback loop. Within this mechanism, inflammation exacerbates oxidative damage, while oxidative stress promotes the release of inflammatory mediators, collectively contributing to hepatocyte injury.

The acidic polysaccharide components of *Lycium ruthenicum* (*L. ruthenicum* polysaccharides, LRP) exhibit significant biological activity, attributed to their unique monosaccharide composition and conformation [11,12]. Current studies have shown that *L. ruthenicum* activates the Nrf2 signalling axis, enhancing the expression of endogenous antioxidant enzymes such as SOD and glutathione peroxidase [13,14]. LRP can also inhibit the TLR4/NF-κB pathway, thereby downregulating the transcription of pro-inflammatory cytokines such as TNF-α and IL-1β [15]. In non-alcoholic fatty liver models, LRP has been shown to alleviate hepatocyte lipid deposition and fibrosis. This synergistic regulation of the oxidative–inflammation network allows LRP to demonstrate unique advantages in intervening in the core pathological processes of liver injury [16], yet its specific protective efficacy in acute liver injury has not been fully elucidated.

This study aimed to elucidate the systemic protective effects of LRP in acute liver injury. First, the physicochemical properties of LRP were characterized by chromatographic techniques. Using classical CCl_4_-induced liver injury models, we quantitatively analysed the dynamic regulation of oxidative-stress markers (SOD/MDA) and inflammatory cytokine profiles (TNF-α/IL-6/IL-10). The restorative effects were evaluated by combining histopathological examination with liver-function indicators. The results provide new insights for developing natural-polysaccharide-based hepatoprotective strategies and expand the application prospects of food-medicine homologous resources in preventing and treating major liver diseases.

## 2. Materials and Methods

### 2.1. Chemicals and Reagents

Fresh *L. ruthenicum Murr.* berries were purchased from Xuelang Agricultural Market, Wuxi, Jiangsu Province, China. Carbon tetrachloride (CCl_4_) was supplied by Shanghai Sangon Biotechnology Co., Ltd. (Shanghai, China). The hematoxylin and eosin (H&E) staining kit, superoxide dismutase (SOD) assay kit and malondialdehyde (MDA) assay kit were obtained from Beijing Solarbio Science & Technology Co., Ltd. (Beijing, China). The cell counting kit-8 (CCK-8, Cat#:C0037) and the Calcein/PI cell viability/cytotoxicity assay kit (Cat#:C2015S) were purchased from Beyotime Biotechnology (Shanghai, China). Enzyme-linked immunosorbent assay (ELISA) kits for interleukin-6 (IL-6, Cat#: EHJ-95903m), interleukin-1β (IL-1β, Cat#: EHJ-30026m), tumor necrosis factor-α (TNF-α, Cat#: EHJ-45111m) and interleukin-10 (IL-10, Cat#: EHJ-47391m) were acquired from Xiamen Huijia Biotechnology (Xiamen, China). Aspartate aminotransferase and alanine aminotransferase (ALT) assay kits were procured from Nanjing Jiancheng Bioengineering Institute. All other reagents were purchased from Sinopharm Chemical Reagent Co., Ltd. (Shanghai, China).

### 2.2. Extraction of LRP

Fresh *L. ruthenicum* berries were weighed at a solid-to-liquid ratio of 1:30 (g/mL) and infused in boiling deionised water for 30 min until fully swollen. The rehydrated berries were then homogenised in a blender, and the slurry was vacuum-filtered; the residue was re-extracted twice under identical conditions, and all filtrates were pooled. The combined filtrate was concentrated to one-third of its original volume under reduced pressure at 60 °C. Four volumes of absolute ethanol were added to reach a final concentration of 80%(*v*/*v*), and the mixture was kept at 4 °C for 12 h. The precipitate was collected by centrifugation, washed successively with absolute ethanol and acetone, and dried to yield the crude polysaccharide. The crude extract was redissolved in distilled water, deproteinised with Sevag reagent (chloroform: n-butanol, 4:1, *v*/*v*) until no protein band was detectable, and dialysed against distilled water for 48 h. The dialysed solution was further concentrated and lyophilised to obtain the purified LRP.

### 2.3. Monosaccharide Composition Analysis

Under a nitrogen atmosphere, 20 mg of LRP was transferred into a sealed 10 mL ampoule, 2 mL of 3 mol/L trifluoroacetic acid (TFA) was added, and the mixture was hydrolysed at 95 °C for 8 h to achieve complete depolymerisation. The monosaccharide composition of the hydrolysate was then analysed according to the protocol of Qin et al. [17]. Briefly, 100 μL of the hydrolysed LRP solution was mixed with 300 μL of 0.3 mol/L NaOH, followed by the addition of 200 μL methanol and 0.5 mol/L 1-phenyl-3-methyl-5-pyrazolone (PMP). The derivatisation was performed at 70 °C for 60 min and terminated with 200 μL of 0.3 mol/L HCl. The reaction mixture was extracted three times with 2 mL chloroform; the aqueous phase was collected, filtered through a 0.45 μm membrane, and subjected to HPLC analysis. Chromatographic separation was carried out on a Galaksil-EF-C18 Bio column (250 mm × 4.6 mm, 5 μm) at 30 °C. Mobile phase A consisted of 0.1 mol/L phosphate buffer (pH 6.8) and mobile phase B was acetonitrile, delivered at a gradient of 83% A/17% B (isocratic). The flow rate was set to 1.0 mL/min and UV detection was performed at 245 nm.

### 2.4. Molecular Weight Analysis

An LRP solution (5 mg/mL) was prepared, and its molecular weight distribution was determined by high-performance liquid chromatography gel filtration chromatography (HPGFC) [18]. The analysis was performed on a Waters 2695 HPLC system equipped with a Waters 2410 refractive-index detector and Empower workstation. The column temperature was maintained at 20 °C; the mobile phase was 0.1 mol/L NaNO_3_ delivered at 0.5 mL/min. A calibration curve was constructed using glucose and a series of dextran standards (T-5, T-10, T-150, T-300, and T-2000) of known molecular weights, and the molecular weight of LRP was calculated accordingly.

### 2.5. FT-IR and UV-Vis Analysis

The structural features of LRP were characterised by Fourier-transform infrared spectroscopy (FT-IR). Exactly 5 mg of the sample was ground with 500 mg potassium bromide (KBr) and pressed into a pellet. The spectrum was recorded from 4000–500 cm^−1^ [19]. To verify the removal of proteins and nucleic acids, a 0.5 mg/mL LRP solution was prepared and its absorbance was measured on a ultraviolet–visible (UV-vis) spectral across 190–400 nm [20].

### 2.6. Cell Viability Assay

The AML12 cell line was purchased from Zhong Qiao Xin Zhou Biotechnology Co., Ltd. (Shanghai, China). Cells were seeded at 1.0 × 10^5^ cells per well in 24-well plates and incubated at 37 °C with 5% CO_2_ until confluence. Hepatotoxicity was induced by exposing the cells to 8 mmol/L carbon tetrachloride (CCl_4_) prepared in serum-free DMEM/F12 medium for 4 h. After injury, the damaged cells were treated with LRP at 30, 60, 90, and 120 μg/mL for 24 h. Cell viability was then assessed using the Calcein/PI Cell Viability/Cytotoxicity Assay Kit to identify the optimal LRP concentration. A second CCl_4_ insult was applied, followed by treatment with the selected optimal concentration of LRP for 24 h. Cells were detached by three freeze–thaw cycles, resuspended in PBS, collected into microcentrifuge tubes, and stored for subsequent analyses.

### 2.7. Animal Experimental Design

The experiment utilized six-week-old healthy male SPF-grade mice. The study protocol was approved by the Animal Care and Ethics Committee of Jiangnan University (Approval JN. No. 20250615c0320715[356]), and all experiments were conducted at the experimental animal centre of Jiangnan University. After one week of acclimatization, the animals were randomly divided into four groups (*n* = 6). Mice were randomly assigned into groups by means of drawing lots. The random allocation was performed by an independent researcher. The sample sizes were determined to ensure the reproducibility of the experiments and in accordance with the 3Rs principles of animal ethics.

(1) Control group: Mice were administered saline daily by oral gavage for 7 consecutive days.
(2) CCl_4_ group: Mice were administered saline daily by oral gavage for 7 consecutive days, followed by a single intraperitoneal injection of 0.3% CCl_4_ solution (10 mL/kg).
(3) 100 mg/kg LRP group: Mice were administered 100 mg/kg LRP daily by oral gavage for 7 consecutive days, followed by a single intraperitoneal injection of 0.3% CCl_4_ solution (10 mL/kg).
(4) 200 mg/kg LRP Mice were administered 200 mg/kg LRP daily by oral gavage for 7 consecutive days, followed by a single intraperitoneal injection of 0.3% CCl_4_ solution (10 mL/kg)

**Figure 1:**
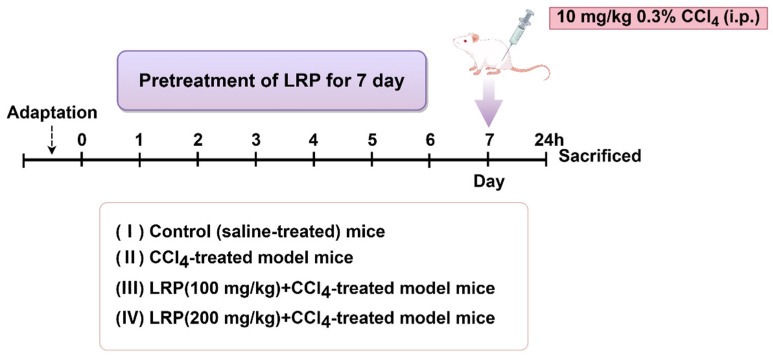
The animal experiment flow chart.

One hour after the final administration, mice received a single intraperitoneal injection of 0.3% carbon tetrachloride (CCl_4_) solution (10 mL/kg body weight). Animals were euthanised 24 h post-injection, with blood samples and hepatic tissues collected for subsequent analysis (Figure 1).

### 2.8. Histopathological Examination

The harvested hepatic tissue was fixed in 4% (*w*/*v*) paraformaldehyde, dehydrated through an ascending ethanol series, cleared in xylene, and embedded in paraffin. Sections of 5 μm thickness were cut and stained with haematoxylin and eosin (H&E). Histopathological changes were examined under a standard inverted light microscope.

### 2.9. Biochemical Examinations of Serum ALT and AST

Serum aspartate aminotransferase (AST) and alanine aminotransferase (ALT) activities were determined using commercial kits from Jiancheng Bioengineering Institute (Nanjing, China) according to the manufacturer’s instructions.

### 2.10. Determination of Body-Weight Changes and Hepatic Index in Mice

Body mass was recorded immediately prior to dissection; the liver was then excised and weighed. Hepatic index was calculated as:

Hepatic index (%) = (liver weight/body weight) × 100.

### 2.11. Assessment of Oxidative Stress Levels

According to the manufacturer’s instructions, Superoxide dismutase (SOD) and malondialdehyde (MDA) levels in cell lysates and mouse liver homogenates were measured using commercial kits.

### 2.12. Determination of Inflammatory Cytokine Levels

Liver samples (50 mg) were washed with ice-cold saline to remove excess blood, then homogenised in 500 μL ice-cold PBS using a tissue grinder. Homogenates were centrifuged at 12,000 rpm for 10 min at 4 °C and the supernatants were collected. IL-1β, IL-6, TNF-α and IL-10 concentrations in cell lysates and liver supernatants were quantified by commercial enzyme-linked immunosorbent assay (ELISA) kits following the manufacturer’s instructions.

### 2.13. Statistical Analysis

GraphPad Prism 9.0 was utilized for all data analysis. The data are presented as mean ± standard deviation (SD). The One-way ANOVA was employed to compare three or more groups. Statistical significance was considered at a level of *p* < 0.05, and the following notations were used: (* *p* < 0.05, ** *p* < 0.01, *** *p* < 0.001, **** *p* < 0.0001). The term “ns” was employed to denote no significant difference.

## 3. Results

### 3.1. Chemical Composition and Structural Characterization Analysis of LRP

**Figure 2:**
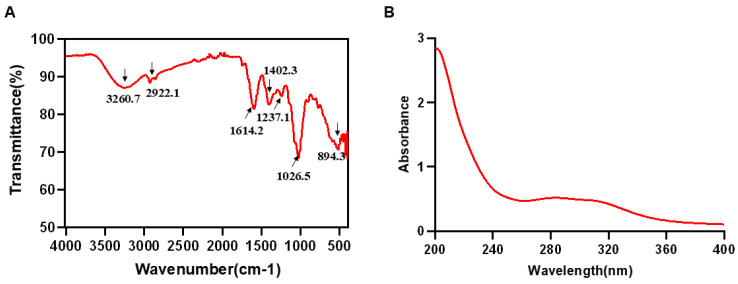
**FT**-**IR and UV**-**vis analysis of LRP.** (**A**) The result of FT-IR spectrum in LRP. (**B**) The result of UV-Vis spectrum in LRP.

The polysaccharide sample obtained by hot water extraction and ethanol precipitation was determined to contain 91.75% polysaccharides and 7.63% proteins. The FT-IR spectrum of LRP is shown in Figure 2A. A broad absorption band at 3260.7 cm^−1^ was assigned to O-H stretching vibrations [21,22], while the weak peak at 2922.1 cm^−1^ corresponded to C-H stretching [23]. The band at 1614.2 cm^−1^ was attributed to the asymmetric stretching vibration of carbonyl (C=O) groups [24]. Peaks at 1402.3 and 1237.1 cm^−1^ arose from C-O stretching and O-H bending, respectively, indicating the presence of carboxylic acid (-COOH) moieties [25]. A characteristic absorption at 1026.5 cm^−1^ suggested a pyranose form, and the signal between 898 and 888 cm^−1^ was consistent with a β-pyranose ring configuration [26]. Furthermore, the UV-Vis spectrum (Figure 2B) revealed no appreciable absorption between 260 and 280 nm, confirming the low contents of protein and nucleic acid impurities in the purified LRP [27,28]. Subsequent HPGFC and monosaccharide analyses showed that LRP is a heteropolysaccharide with a weight-average molecular mass of 673 Da. It was composed primarily of glucose 58.42%, arabinose 14.99%, galactose 9.26%, glucuronic acid 4.49%, fucose 3.63%, mannose 2.61%, rhamnose 2.25%, xylose 1.90%, galacturonic acid 1.56% and fructose 0.89% (Table 1).

### 3.2. LRP Improved Cell Viability, Inflammation and Oxidative Stress of Hepatocytes

**Figure 3:**
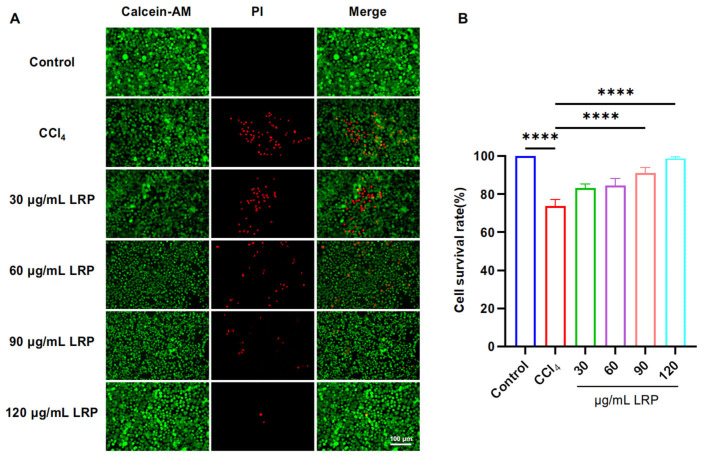
**LRP improved the activity of liver cells.** (**A**) Live/dead staining results of AML-12 cells treated with different concentrations of LRP. (**B**) Quantitative analysis of live/dead staining (*n* = 3); **** *p* < 0.0001.

To determine the optimal intervention concentration of LRP, we evaluated its protective effect against CCl_4_-induced liver injury using a live/dead cell staining assay (Figure 3). The results showed that pre-treatment with 90 µg/mL and 120 µg/mL LRP significantly increased the hepatocyte viability to 91.16 ± 2.90% and 98.82 ± 0.75%, respectively, compared to the injury model group (73.83 ± 3.40%) (*p* < 0.0001). Based on these findings, 90 µg/mL and 120 µg/mL were selected as the low and high doses for subsequent studies.

**Figure 4:**
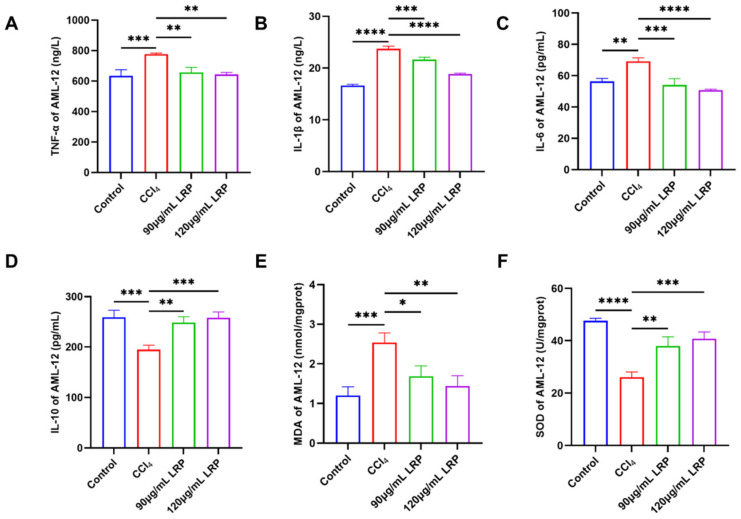
**LRP ameliorated hepatic inflammation and oxidative stress.** (**A**–**D**) The levels of TNF-α, IL-1β, IL-6, and IL-10 in AML-12 cells (*n* = 3). (**E**,**F**) Levels of MDA and activity of SOD in AML-12 cells (*n* = 3); * *p* < 0.05, ** *p* < 0.01, *** *p* < 0.001, **** *p* < 0.0001.

The anti-inflammatory potential of LRP was evaluated by quantifying the pro-inflammatory cytokines TNF-α, IL-1β and IL-6 together with the anti-inflammatory cytokine IL-10 (Figure 4A–D). At 120 µg/mL LRP, the expression of TNF-α, IL-1β and IL-6 was reduced to 17.23%, 20.58% and 26.50% of the model group values, respectively (*p* < 0.01, *p* < 0.0001). Concurrently, IL-10 levels rose from 194.88 ± 8.63 pg/mL in the model group to 248.37 ± 11.91 pg/mL at 90 µg/mL LRP and 257.79 ± 11.78 pg/mL at 120 µg/mL LRP, representing increases of 21.54% and 24.40% (*p* < 0.001), indicating a marked improvement in the hepatic inflammatory milieu. Further analysis of oxidative stress markers revealed (Figure 4E,F): The lipid peroxidation product MDA exhibited significant reductions of 33.48% (1.69 ± 0.26 nmol/mg prot) and 43.30% (1.44 ± 0.26 nmol/mg prot) in the low- and high-dose groups, respectively, compared to the model group (2.54 ± 0.24 nmol/mg prot; *p* < 0.05, *p* < 0.01).

### 3.3. LRP Repaired CCl_4_-Induced Liver Injury of Mice

**Figure 5:**
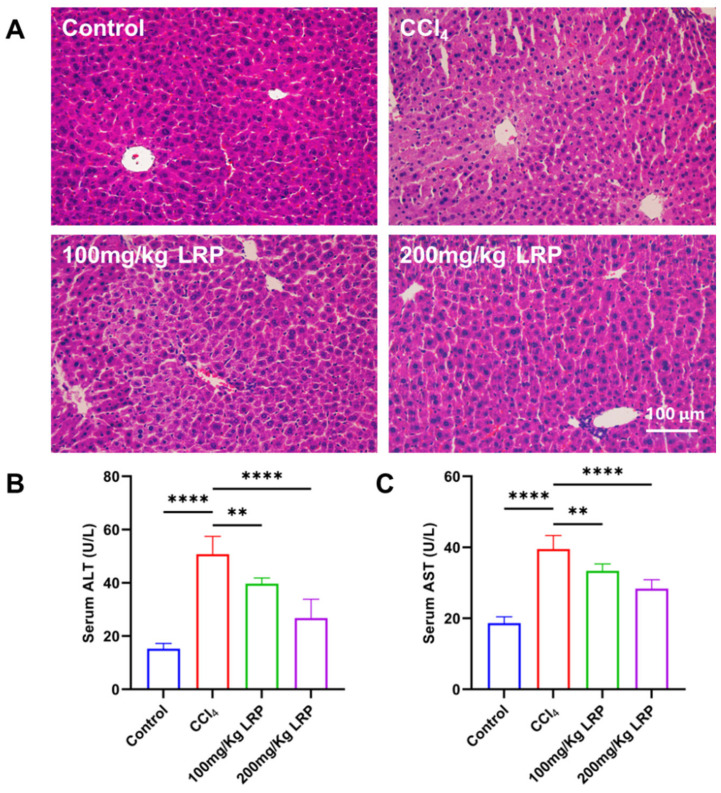
**LRP promoted histopathological restoration in the liver of mice with CCl_4_-induced injury.** (**A**) Histological analysis of mice liver tissues by H&E staining. (**B**,**C**) Measurement of serum ALT and AST levels (*n* = 6); ** *p* < 0.01, **** *p* < 0.0001.

Histopathological examination of H&E-stained sections (Figure 5A) revealed that hepatocytes in the control group exhibited regular morphology and orderly architecture. In contrast, the model group displayed characteristic pathological damage: marked disarray around the portal areas, pale and swollen cytoplasm, extensive nuclear pyknosis and karyorrhexis, and focal karyolysis. After LRP administration, both low- and high-dose groups showed pronounced attenuation of these lesions; hepatocyte integrity was restored, cytoplasmic density and nuclear morphology approached normal values, and necrotic areas were virtually absent. Biochemical analyses (Figure 5B,C) demonstrated that serum ALT and AST activities in the model group (50.75 ± 6.70 U/L and 39.52 ± 3.80 U/L, respectively) were 70.01% and 52.85% higher than those in the control group (ALT 15.23 ± 2.03 U/L; AST 18.63 ± 1.77 U/L) (*p* < 0.0001). Following LRP intervention, ALT and AST decreased to 39.68 ± 2.18 U/L and 33.36 ± 1.96 U/L in the low-dose group and further declined to 26.75 ± 7.07 U/L and 28.35 ± 2.52 U/L in the high-dose group. During the study, no deaths occurred among the mice in either the control group, model group or the LRP-treated group.

The liver tissues of CCl_4_-induced mice evoked hepatic oedema and inflammatory infiltration, reflected by marked increases in hepatic wet weight and the corresponding hepatic index. Quantitative data (Table 2) showed that, relative to control (0.88 ± 0.03 g, 4.25 ± 0.14%), the model group exhibited rises of 7.82% and 13.83% in hepatic wet weight (0.96 ± 0.06 g) and hepatic index (4.93 ± 0.27), respectively (*p* < 0.01). LRP administration dose-dependently reversed these elevations: the low-dose cohort (100 mg/kg) reduced the hepatic index to 4.53 ± 0.24%, whereas the high-dose cohort (200 mg/kg) further decreased it to 4.32 ± 0.27% (*p* < 0.01). Both hepatic wet weight and hepatic index declined progressively with increasing LRP dose, indicating a dose-dependent protective effect.

### 3.4. LRP Attenuated Inflammation and Oxidative Stress of Liver Tissue

**Figure 6:**
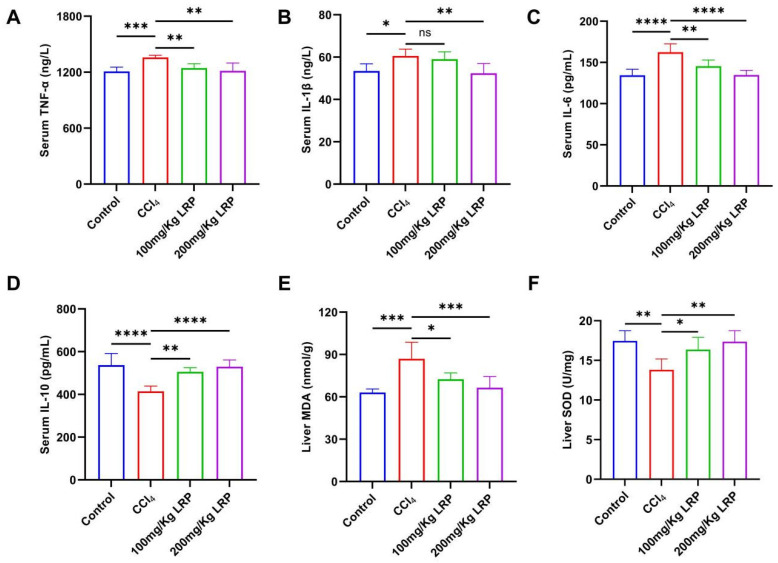
**LRP alleviated inflammation and oxidative stress in mice with hepatic injury.** (**A**–**D**) The contents of TNF-α, IL-1β, IL-6, and IL-10 in the serum of mice (*n* = 6). (**E**,**F**) Levels of MDA and SOD in liver tissues (*n* = 6); * *p* < 0.05, ** *p* < 0.01, *** *p* < 0.001, **** *p* < 0.0001.

At the inflammatory level, the model group exhibited 1.1, 1.2 and 1.1 times increases in the pro-inflammatory cytokines TNF-α, IL-6 and IL-1β, respectively, relative to the control group. High-dose LRP treatment reversed these elevations by 10.58%, 17.00% and 13.50% (*p* < 0.01, *p* < 0.0001) and concurrently enhanced IL-10 by 21.82% (*p* < 0.0001). Notably, low-dose LRP did not significantly modulate IL-1β (59.04 ± 3.44 pg/mL vs. 60.56 ± 3.23 pg/mL, *p* > 0.05), underscoring the dose-dependent nature of its anti-inflammatory action. Furthermore, CCl_4_ induction significantly increased hepatic MDA levels by 27.42% compared to the control group (*p* < 0.001), while it inhibited SOD activity, which decreased by 20.92% (*p* < 0.01), indicating lipid peroxidation damage and an imbalance in antioxidant defence. LRP intervention produced a dose-dependent protective response: at 200 mg/kg, MDA declined by 23.51% to 66.49 ± 7.97 nmol/g protein and SOD activity rose by 20.42% to 17.36 ± 1.38 U/mg protein (*p* < 0.001, *p* < 0.01), whereas the 100 mg/kg dose also significantly improved both parameters (*p* < 0.05) (Figure 6).

## 4. Discussion

The study was centrally concerned with delineating the hepatoprotective potential and mechanistic basis of LRP in acute liver injury. Integrating in vivo and in vitro approaches, we provided the first unequivocal demonstration that LRP, at precisely defined doses, effectively attenuated CCl_4_-induced hepatic damage. Specifically, LRP markedly enhanced hepatocyte viability, substantially reduced serum ALT and AST activities, and significantly ameliorated histopathological lesions, thereby furnishing direct and robust empirical evidence for its hepato-protective efficacy.

The protective effect of LRP primarily relies on its anti-inflammatory and antioxidant activities. LRP markedly enhanced endogenous SOD activity and concomitantly suppressed MDA generation, underscoring its potent antioxidant capacity. Simultaneously, LRP down-regulated the key pro-inflammatory cytokines (TNF-α, IL-1β, IL-6) while up-regulating the anti-inflammatory mediator IL-10, indicating a systematic rebalancing of the hepatic inflammatory milieu. This coordinated antioxidant and anti-inflammatory intervention appeared to interrupt the positive feedback loop between oxidative stress and inflammation-excessive ROS can activate inflammasome-related pathways (e.g., NLRP3), whereas amplified inflammation further exacerbates oxidative damage [29,30]. LRP may therefore exert its effects by disrupting this cycle. This coordinated antioxidant and anti-inflammatory regulation constitutes the core mechanism underlying the protective effect of LRP. Our recent study revealed that LRP alleviates lipopolysaccharide (LPS)-induced acute lung injury through its anti-inflammatory properties and by restoring gut microbiota homeostasis [31]. Polysaccharides have been reported to exert comparable mechanisms in inflammatory diseases. For example, previous studies have shown that cordyceps polysaccharides can similarly alleviate immune responses and inflammatory damage in acute liver failure (ALF) by modulating the balance between pro-inflammatory and anti-inflammatory factors and reducing cell apoptosis [32], which bears a certain resemblance to the mechanism of LRP. Moreover, studies have shown that polysaccharides derived from *L. ruthenicum* not only possess antioxidant and anti-inflammatory activities but also indirectly influence hepatic function through immune system modulation. For instance, *L. ruthenicum* polysaccharide 3 has been reported to significantly increase the spleen index in cyclophosphamide-treated mice, enhance the proliferative responses of T cell and B cell, and improve the phagocytic activity of peritoneal macrophages, thereby counteracting the immunosuppressive effects on immune organ development [33]. Notably, as a central organ in immune regulation, the spleen is closely connected to the liver through the portal venous system. Recent research has revealed that obesity and metabolic stress can induce immune cell imbalance between the liver and spleen, a form of “inter-organ crosstalk” that often exacerbates hepatic oxidative stress and inflammatory responses [34].

Notably, the molecular characteristics of LRP (molecular weight: 673 Da, containing β-pyranose rings, with glucose units comprising 58.42% of monosaccharide composition) may be fundamentally linked to its bioactivity. β-Glucans with defined conformations can, in principle, interact with pattern-recognition receptors such as dectin-1 to modulate intracellular signalling [35,36]. However, we did not experimentally investigate receptor binding or downstream cascades; these mechanistic hypotheses warrant future validation.

Several limitations merit acknowledgment. Due to the inherent nature of the experimental intervention, it was not possible to blind either the investigators or the mice to group allocation, which may introduce a potential risk of bias. Nevertheless, blinding was applied to the outcome assessors to minimize subjective bias, although some degree of systematic error could not be completely avoided. Future studies should carefully address these methodological issues at the design stage to ensure the reliability and consistency of the results. In addition, while the in vitro cell model provided important mechanistic insights, it may not fully recapitulate the complexity of in vivo conditions. The biological mechanisms observed in this study, such as the modulation of oxidative stress and inflammatory responses, are highly conserved among mammalian species, suggesting that these findings may offer valuable implications for similar pathophysiological processes in humans. Nonetheless, further investigations in higher animal models and clinical settings are needed to validate the translational relevance and long-term efficacy of LRP, as well as to elucidate its specific molecular mechanisms in protecting against acute liver injury.

Collectively, our findings provide both theoretical and practical significance. LRP appears to orchestrate a synergistic hepatoprotective network by integrating anti-inflammatory and antioxidant responses. As a naturally abundant and readily obtainable polysaccharide, LRP holds considerable promise for the repair of acute liver injury and offers a unique avenue for the development of novel therapeutic strategies against liver diseases.

## 5. Conclusions

Taken together, the study confirmed that LRP, a heteropolysaccharide of 673 Da, markedly alleviated CCl_4_-induced ALI through dual antioxidant and anti-inflammatory mechanisms. In vitro, LRP increased hepatocyte viability, decreased MDA, elevated SOD, and suppressed TNF-α, IL-1β and IL-6 while enhancing IL-10. In vivo, LRP improved hepatic histopathology, down-regulated serum transaminases, and promoted hepatic repair. These findings highlighted the novel potential of LRP as a natural polysaccharide-based agent for acute liver injury protection.

## Figures and Tables

**Table 1:** The Monosaccharide composition of LRP.

Types of Monosaccharides	Percentage (%)
Glucose	58.42
Arabinose	14.99
Galactose	9.26
Glucuronic acid	4.49
Fucose	3.63
Mannose	2.61
Rhamnose	2.25
Xylose	1.90
Galacturonic acid	1.56
Fructose	0.89

**Table 2:** The analysis of hepatic index of mice.

	Liver Wet Weight (g)	Body Mass(g)	Hepatic Index (%)
Control	0.88 ± 0.03	20.79 ± 0.64	4.25 ± 0.14
CCl_4_	0.96 ± 0.06 *	19.43 ± 0.71 **	4.93 ± 0.27 **
100 mg/kg LRP	0.93 ± 0.03	20.52 ± 0.59 ^#^	4.53 ± 0.24 ^#^
200 mg/kg LRP	0.89 ± 0.06 ^#^	20.68 ± 0.39 ^#^	4.32 ± 0.27 ^##^

Note: * *p* < 0.05, ** *p*< 0.01 versus normal control; ^#^ *p* < 0.05, ^##^ *p* < 0.01 versus model group (*n* = 6).

## Data Availability

The datasets generated during and/or analysed during the current study are available from the corresponding author upon reasonable request. However, the data are currently not publicly available due to pending patent application.
